# Optimization of primer sets and detection protocols for SARS-CoV-2 of coronavirus disease 2019 (COVID-19) using PCR and real-time PCR

**DOI:** 10.1038/s12276-020-0452-7

**Published:** 2020-06-16

**Authors:** Myungsun Park, Joungha Won, Byung Yoon Choi, C. Justin Lee

**Affiliations:** 1 Center for Cognition and Sociality, Cognitive Glioscience Group, Institute for Basic Science, Daejeon, 34126 Republic of Korea; 2 Department of Biological Sciences, Korea Advanced Institute of Science and Technology (KAIST), Daejeon, 34141 Republic of Korea; 3 Department of Otorhinolaryngology, Seoul National University Bundang Hospital, Seongnam, 13620 Korea

**Keywords:** Viral infection, Disease genetics

## Abstract

SARS-CoV-2 is very contagious and has rapidly spread globally. Due to various symptomatic and asymptomatic cases and the possibility of asymptomatic transmission, there is a pressing need for a fast and sensitive detection protocol to diagnose asymptomatic people. Various SARS-CoV-2 diagnostic kits are already available from many companies and national health agencies. However, publicly available information on these diagnostic kits is lacking. In response to the growing need and the lack of information, we developed and made available a low-cost, easy-access, real-time PCR-based protocol for the early detection of the virus in a previous study. During the development of the detection protocol, we found that unoptimized primer sets could inadvertently show false-positive results, raising the possibility that commercially available diagnostic kits might also contain primer sets that produce false-positive results. Here, we provide three-step guidelines for the design and optimization of specific primer sets. The three steps include (1) the selection of primer sets for target genes (*RdRP*, *N*, *E*, and *S*) in the genome of interest (SARS-CoV-2), (2) the in silico validation of primer and amplicon sequences, and (3) the optimization of PCR conditions (i.e., primer concentrations and annealing temperatures) for specific hybridization between the primers and target genes, and the elimination of spurious primer dimers. Furthermore, we have expanded the previously developed real-time PCR-based protocol to more conventional PCR-based protocols and applied a multiplex PCR-based protocol that allows the simultaneous testing of primer sets for *RdRP*, *N*, *E*, and *S* all in one reaction. Our newly optimized protocol should be helpful for the large-scale, high-fidelity screening of asymptomatic people, even without any high-specification equipment, for the further prevention of transmission, and to achieve early intervention and treatment for the rapidly propagating virus.

## Diagnostic microbiology: Boosting confidence in COVID-19 detection

A design strategy for virus detection tests could lead to improved detection of SARS-CoV-2 coronavirus. Laboratories currently perform SARS-CoV-2 diagnostic assays based on a technique called the polymerase chain reaction (PCR), which enables targeted amplification of viral nucleic acids. PCR assays are sensitive but can be plagued by false positives or false negatives. C. Justin Lee of the Institute for Basic Science, Daejon, Republic of Korea, and coworkers have devised a protocol for optimizing assay performance. The success of a PCR assay is determined by the selection of ‘primers’, short DNA strands that determine which genomic sequence gets amplified. The researchers identify a strategy for designing effective primer sets, and selecting reaction conditions that make best use of those primers. Although the focus here is on SARS-CoV-2, this approach should be applicable to other viruses.

## Introduction

Severe acute respiratory syndrome coronavirus 2 (SARS-CoV-2) is a positive-sense single-stranded RNA virus^1^. It is the viral strain that causes coronavirus disease 2019 (COVID-19), whose major symptoms include severe pneumonia in humans^2^. In addition to pneumonia, other symptoms include high fever, dry cough, fatigue, and frequent pain in the digestive system^3^. The World Health Organization (WHO) has designated the ongoing pandemic of COVID-19 as a Public Health Emergency of International Concern^2^. As of April 11, 2020, COVID-19 affects 210 countries, and the estimated case number has reached 1,700,000 people, with a death toll of over 100,000 people, while the number of recovered patients is approximately 382,000 people^4^. Due to the spread of the pandemic, there is a desperate need for the development of diagnostic and treatment tools for COVID-19. Various SARS-CoV-2 diagnostic kits are already available from many companies and national health agencies. Most of these kits are based on real-time polymerase chain reaction (real-time PCR) technology. However, publicly available information on these diagnostic kits is lacking.

COVID-19 is transmitted from person to person and is propagated primarily via respiratory drops released through coughing and sneezing within a range of ~1.8 meters (6 feet)^5,6^. The extent of virus transmission during the incubation period is uncertain, but studies have shown that the pharynx shows its peak viral load approximately 4 days after infection^7,8^. More alarmingly, numerous cases of asymptomatic transmission (transmission of the virus by a virus-carrying person before they show any symptoms) have been reported^9^. The virulence of SARS-CoV-2 can be estimated according to the reproduction number (*R*_0_)^10^. *R*_0_ is a measurement of how many people a contagious person transmits the virus to. The WHO has estimated the basic *R*_0_ of SARS-CoV-2 to be between 1.4 and 2.5^10^. However, according to Swedish researchers, the average *R*_0_ is 3.28, and the maximum is 6.49^10^, which are much higher values than the estimates of the WHO. More recently, Sanche et al. reported a median R_0_ value of 5.7 (95% confidence interval 3.8–8.9)^11^. The ability to spread SARS-CoV-2 is predicted to be similar to or higher than that of the previous SARS-CoV (SARS), which showed *R*_0_ values ranging between 2 and 5^12^. The highly infectious nature of SARS-CoV-2 calls for the rapid distribution of detection protocols, diagnostic tools, and treatment options.

Currently, various SARS-CoV-2 diagnostic kits are available from many companies and national health agencies. However, there are many obvious limitations to the current diagnostic methods. Many companies have developed and sold diagnostic kits hurriedly and hastily as the demand has soared, without proper validation or extensive testing. In fact, China and the United States reported problems with the reliability of the available test kits at the beginning of the COVID-19 outbreak in each country^13,14^. The Czech Republic reported incorrect results for ~80% of the test kits purchased from China^15^. In our recent report, we have demonstrated the possibility of obtaining a false-positive result when primers are not verified during the development of primer sets for the detection of SARS-CoV-2 through real-time PCR^9^. A similar report appeared in a medical archive, indicating the possibility of a false positive when the authors compared the nine previously published primer sets for SARS-CoV-2^16^. To make matters worse, many commercially available kits do not publicly disclose the critical sequence information of the primer sets that they contain, making it impossible to carefully verify and validate the quality and sensitivity of each primer set.

In response to the growing need and lack of information, we developed and made available a low-cost, easy-access, real-time PCR-based protocol for early detection of the virus in a previous study^9^. During the development of the detection protocol, we found that unoptimized primer sets could inadvertently show false-positive results^9^. To address this issue, we set out to develop simple, clear guidelines for designing and optimizing a primer set. In addition, we expanded the previously developed real-time PCR-based protocol to a more conventional PCR-based protocol and applied a multiplex PCR-based protocol to facilitate the broad availability and easy accessibility of the protocol.

## Materials and methods

### Volunteer recruitment

The purpose of sampling and the procedure involving the use of a pharyngeal swab were approved by the Seoul National University Hospital Institutional Review Board (IRBY-H-1807-197-966) and thoroughly explained to each volunteer. We received written consent from the volunteer. A total of one volunteer participated in this experiment (Volunteer U).

### Pharyngeal swab procedure

The detailed pharyngeal swab procedure was modified from the previously described procedure^9^. Briefly, we used a polyester swab with a plastic shaft (6.4 × 3.4 × 16.8-mm tip, 163.3-mm length, Catalog #: 6-6587-31, LMS, Japan) rather than a wood shaft. After sample collection, the swab was immediately placed in a 1.5-ml microcentrifuge tube (Axygen® 1.5 mL MaxyClear Snaplock Microcentrifuge Tube, Polypropylene, Clear, Nonsterile, Catalog #: MCT-150-C, Axygen, USA). The collected sample on the swab was then immediately transferred to and dissolved into 200 μl of TRIzol (easy-BLUE™ Total RNA Extraction Kit, Catalog #17061, iNtRON, Republic of Korea) by vigorously mixing with swirling during which the swab was passed up and down at least 20 times. Then, an additional 500 μl of TRIzol was added to a 1.5-ml microcentrifuge tube, and mixing was performed by inverting the tube five times.

### HEK-293T cell culture for the positive control

The HEK-293T cell line (ATCC, Lot # 70008735) was used as a positive control for human tissue. HEK-293T cells are a commonly used human cell line derived from the human embryonic kidney-293 (HEK-293) cell line that expresses a mutant version of the SV40 large T antigen^17^. These cells were cultured in Dulbecco’s modified Eagle’s medium (DMEM, Catalog #: 10-013-CV, Corning, USA) supplemented with 10% fetal bovine serum (fetal bovine serum, certified, heat inactivated, Catalog #: 1008214, Thermo Fisher Scientific, USA) and 1% penicillin and streptomycin (HyClone™ Penicillin–Streptomycin Solution (100×), Catalog #: Sv30010, GE Healthcare, USA). The cells were maintained in a 37 °C humidified atmosphere in a 5% CO_2_ incubator. Finally, 700 μl of TRIzol was added to the HEK-293T cell pellet for RNA extraction.

### RNA extraction

The total RNA of the sample collected from the volunteer and HEK-293T cells (~1 × 10^7^ cells) were extracted by using the TRIzol reagent in a 1.5-ml microcentrifuge tube as previously described^9^. Each sample was incubated in TRIzol for 5 min at room temperature, 200 μl of chloroform was added, mixing was performed by inverting the tube 5 times, and the tube was incubated for 3 min and centrifuged for 15 min at 12,000 × *g* at 4 °C. The clear upper aqueous layer, which contained RNA, was transferred to a new 1.5-ml tube, and 350 μl of isopropanol was added. After incubation for 10 min on ice, gentle mixing by inverting the tube five times was performed. The sample was centrifuged for 10 min at 12,000 × *g* at 4 °C. The supernatant was discarded, and the remaining pellet was washed in 500 μl of 70% ethanol. The sample was again centrifuged for 10 min at 12,000 × *g* at 4 °C. The supernatant was again discarded, and the RNA pellet was air-dried for 5 min. To solubilize the RNA pellet, the pellet was resuspended in 10 μl of RNase-free water. The RNA concentration and purity were determined with a NanoDrop spectrophotometer (Thermo Fisher Scientific, USA) by calculating the ratio of the optical density at wavelengths of 260 nm/280 nm and 260 nm/230 nm.

### SARS-CoV-2 RNA as a positive control

The positive control containing SARS-CoV-2 viral RNA was obtained from the Korea Centers for Disease Control and Prevention (http://www.cdc.go.kr/). A detailed description of how the SARS-CoV-2 viral RNA was prepared is provided in a separate report^18^. Briefly, SARS-CoV-2 viral RNAs were prepared by extracting total RNA from Vero cells infected with a viral clone, BetaCoV/Korea/KCDC03/2020, at an MOI of 0.05. The Vero cell line was originally isolated from kidney epithelial cells extracted from an African green monkey (*rhesus macaque*)^19^.

### Reverse transcription

RNA was converted into complementary DNA (cDNA) using the SuperScript™ III First-Strand Synthesis System (Catalog #: 18080051, Invitrogen™, USA) following the manufacturer’s recommended procedures with some modifications. Eight microliters of RNA was used for cDNA synthesis, and 200 U of SuperScript III, 1 μl of 50 μM oligo (dT)_20_, 1 μl of 50 ng/µl random hexamers, 1 μl of 10 mM dNTPs, and 9 μl of cDNA synthesis mix were added to the reaction. The 20-μl cDNA synthesis reaction mixture was incubated at 25 °C for 10 min and 55 °C for 1 h, followed by an inactivation step at 80 °C for 5 min.

### Primer design

The full sequence of SARS-CoV-2 from the first patient in the Korean reference sequences was retrieved from the NCBI Reference Sequence Database (https://www.ncbi.nlm.nih.gov/nuccore/NC_045512)^20^. To design the primer sets, we utilized the Primer3 tool (http://primer3.wi.mit.edu)^21^. In silico primer tests were performed on two websites, idtdna.com and genome.ucsc.edu. At idtdna.com (https://sg.idtdna.com/pages/tools/oligoanalyzer), we predicted the secondary structure of the primers and the amplicon and the self- and heterodimerization tendencies of each primer set. We performed in silico PCR at the genome.ucsc.edu site (https://genome.ucsc.edu/cgi-bin/hgPcr). The primer sets were synthesized and delivered by Cosmogenetech (Seoul, Republic of Korea). The primer sets designed and used in this study are listed in Table 1.

**Table 1:** Primer list for the optimized SARS-CoV-2 detection protocol.

Target genome	Experiment	Target gene	Primer name	Forward primer (5′–3′)	Reverse primer (5′–3′)	Size (bp)
SARS- CoV-2	Real-time and traditional PCR	RdRP	SARS-CoV-2_IBS_RdRP2^9^	AGAATAGAGCTCGCACCGTA	CTCCTCTAGTGGCGGCTATT	101
		S	SARS-CoV-2_IBS_S2^9^	GCTGGTGCTGCAGCTTATTA	AGGGTCAAGTGCACAGTCTA	107
		N	SARS-CoV-2_IBS_N1^9^	CAATGCTGCAATCGTGCTAC	GTTGCGACTACGTGATGAGG	117
	Real-time, traditional, and multiplex PCR	E	SARS-CoV-2_IBS_E2^9^	TTCGGAAGAGACAGGTACGTTA	AGCAGTACGCACACAATCG	116
	Traditional multiplex PCR	RdRP	SARS-CoV-2_IBS_m_RdRP 1	GCTCGCAAACATACAACGTG	CATTAACATTGGCCGTGACA	202
		RdRP	SARS-CoV-2_IBS_m_RdRP 2	TGAAATCAATAGCCGCCACT	TGTTTGCGAGCAAGAACAAG	199
		S	SARS-CoV-2_IBS_m_S 1	CAGATGCTGGCTTCATCAAA	GGTTGGCAATCAATTTTTGG	291
		S	SARS-CoV-2_IBS_m_S 2	ACTGTTTTGCCACCTTTGCT	AGCTTGTGCATTTTGGTTGA	300
		N	SARS-CoV-2_IBS_m_N 1	AAGGAAATTTTGGGGACCAG	GAGTCAGCACTGCTCATGGA	399
		N	SARS-CoV-2_IBS_m_N 2	AAAGGCCAACAACAACAAGG	GCTCTGTTGGTGGGAATGTT	393
*Homo sapiens*	Real-time and traditional PCR	GAPDH	GAPDH^26^	CAATGACCCCTTCATTGACC	TTGATTTTGGAGGGATCTCG	159
	Traditional multiplex PCR	18S rRNA	18S rRNA^27^	CGGCTACCACATCCAAGGAA	GCTGGAATTACCGCGGCT	186
		ACTB	IBS_m_ACTB 1	CTCCTGAGCGCAAGTACTCC	GTCACCTTCACCGTTCCAGT	299
		ACTB	IBS_m_ACTB 2	AGAGCTACGAGCTGCCTGAC	AGTACTTGCGCTCAGGAGGA	300
		TBP	IBS_m_TBP 1	GTTCTGGGAAAATGGTGTGC	GGAGGCAAGGGTACATGAGA	401
		TBP	IBS_m_TBP 2	AATATGGTGGGGAGCTGTGA	GGCACTTACAGAAGGGCATC	403

List of primers used for PCR and real-time PCR.

### Primer testing through PCR

The accuracy and efficiency of each primer set were verified through PCR amplification of the positive control to optimize the PCR conditions. For each reaction, 5 ng of SARS-CoV-2 cDNA was used for the SARS-CoV-2-specific target primer set, and 5 ng of HEK-293T cDNA was used for the human internal positive control (IPC) primer set. Gradient PCR was performed with an increasing annealing temperature (*T*_a_) from 50 °C to 65 °C based on the melting temperature (*T*_m_) of each primer set. The basic PCR conditions were set according to the protocol provided in the manufacturer’s recommended procedures for each reagent used. We used a premixed PCR reagent to minimize the side effects such as contamination. The recommended optimal concentration range of the primers was 100–500 nM.

### PCR test for cDNA quality

As an optional step, a cDNA quality test was performed after cDNA synthesis to verify the appropriate synthesis of the cDNA from each sample. A GAPDH primer set (Table 1) was used for the human IPC. In this PCR protocol, 5 ng of cDNA was used as a template with the following PCR cycling conditions: 94 °C for 3 min, 35 cycles of 94 °C for 30 s, 62 °C for 40 s, and 72 °C for 1 min, with the final elongation step at 72 °C for 5 min. Each primer was used at a concentration of 500 nM in 2× PCR premix reagent (Quest Taq PCR Mix, Catalog #: QM13532, BioQuest, USA). The amplicons were subjected to electrophoresis in a 2% agarose gel at 130 V for 20 min and visualized using a safe nucleic acid staining solution, LUMI-Green (Nexbio, Daejeon, Republic of Korea), under UV light.

### Traditional PCR for SARS-CoV-2 detection

The SARS-CoV-2_IBS_E2, SARS-CoV-2_IBS_RdRP2, SARS-CoV-2_IBS_S2, and SARS-CoV-2_IBS_N1 primer sets were used for the detection of SARS-CoV-2 transcripts. In addition, the GAPDH primer set was used for the human IPC sample. All PCR conditions were the same as those for the cDNA-quality test PCR procedure.

### Multiplex PCR for SARS-CoV-2 detection

The SARS-CoV-2_IBS_E2, SARS-CoV-2_m_IBS_RdRP 1, SARS-CoV-2_IBS_m_S 1, and SARS-CoV-2_IBS_m_N 1 primer sets were used for the multiplex PCR-based detection of the SARS-CoV-2 transcript. The total concentration of all primer mixes was 500 nM, and the concentration of each set was 125 nM. The IBS_m_ACTB 1, IBS_m_TBP 1, and 18 S rRNA primer sets were used as human IPCs. The final concentration of the primer mix was 500 nM, and the concentration of each set was 167 nM. The PCR conditions were the same as those described above.

### Real-time and multiplex real-time PCR

Five to 10 ng of cDNA and 2X Power SYBR® Green PCR Master Mix (Catalog #: 4368577, Thermo Fisher Scientific, USA) were used. The thermal cycling conditions in the Quantstudio 1 Real-Time PCR system (Applied Biosystems, USA) were 50 °C for 2 min, 95 °C for 10 min, 40 cycles of 95 °C for 15 s, and 62 °C for 1 min, and by a melting curve stage of 95 °C for 10 s and 60 °C for 1 min. The SARS-CoV-2_IBS_E2, SARS-CoV-2_IBS_RdRP2, SARS-CoV-2_IBS_S2, and SARS-CoV-2_IBS_N1 primer sets were used for the real-time PCR-based detection of SARS-CoV-2. In addition, GAPDH primers were used for each sample as the human IPC. The final concentration of the primer mix was 500 nM.

## Results

### Primer design and optimization guidelines

**Fig. 1:**
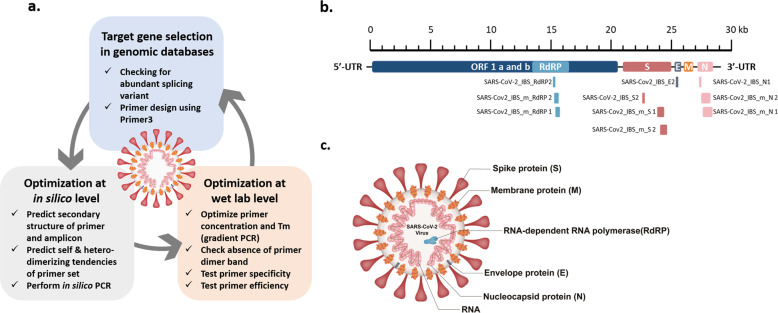
PCR primer design and optimization guidelines, SARS-CoV-2 genome map, and targets of primer sets. **a** Three-step guidelines for PCR primer design and optimization. Step 1: target genes were selected from genomic databases, and primers were designed using Primer3. Step 2: the primer sets were optimized in silico to avoid secondary structure in the primers or untargeted amplification. Step 3: the designed primers were optimized at the wet-lab level to achieve a high specificity and efficiency for detecting the targets. **b** Location of target genes and the selected primer sets in the SARS-CoV-2 genome. **c** The structure of SARS-CoV-2, showing each protein and its name.

In a previous study, we recognized the importance of the process of verifying and optimizing the primer sets used for virus testing. Therefore, in this study, we first describe the detailed guidelines for designing and optimizing the primer sets in three important steps (Fig. 1a). The first step is to select the target genes (*RdRP*, *N*, *E*, and *S*) to be detected in the genome of interest (SARS-CoV-2) and design a primer based on the target sequence of each gene. For an optimal target sequence, when there is a splicing variant in a gene transcript, a target region in the more abundant splicing variant is preferred. For the actual primer design, various tools available at the Primer3 website (http://primer3.wi.mit.edu) were used. Primer3 allows the selection of the optimal primer on the basis of *T*_m_, primer length, and 3′-end stability, which should be considered when designing each primer set. The second step is the in silico validation of primer and amplicon sequences. The identification of amplicon secondary structure and the possibility of self- or heterodimer formation by the primer sequence itself were predicted at another website, idtdna.com (https://sg.idtdna.com/pages/tools/oligoanalyzer). At this website, the tendency for self- or heterodimerization can be estimated by calculating the ΔG value. Finally, at the gemone.ucsc.edu website (https://genome.ucsc.edu/cgi-bin/hgPcr), the in silico PCR tool can be used to predict the possibility of nonspecific reactions in the same genome as well as the genomes of different species. The third step is to experimentally confirm and optimize the primer set in a biological laboratory. We optimized PCR conditions such as the annealing temperature (*T*_a_) through gradient PCR by setting the annealing temperatures between 50 °C and 65 °C and identifying the temperature at which the highest amplification was achieved. As a cautious measure, it is important to note that the final concentration of the primer set in the PCR mixture is critical for target-specific PCR. At concentrations greater than the optimal concentration, primers can form dimers and interfere with target-specific PCR. Therefore, for each primer set, concentrations ranging from 100 nM to 500 nM need to be tested for the formation of dimers. When confirming the PCR result with electrophoresis, the efficiency of the reaction needs to be checked based on whether the band size of the amplicon and the amount of template added are appropriate or not.

**Fig. 2:**
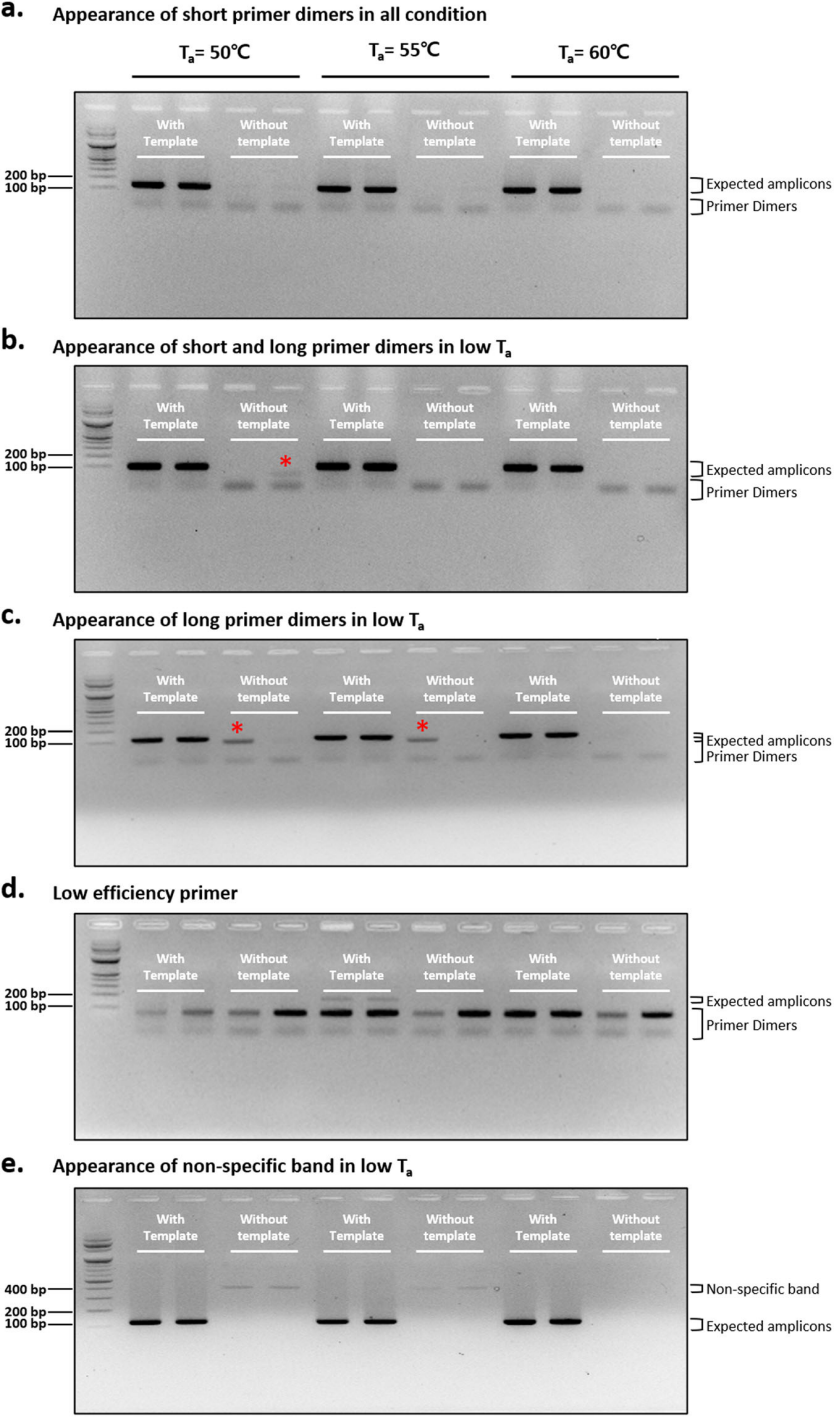
Examples of nonoptimal primer set test results using PCR. Electrophoresis results after PCR (35 cycles) with various nonoptimal primer sets with or without template DNA (SARS-CoV-2 cDNA) and under different *T*_a_ conditions. **a** An example of the appearance of short primer dimers in all conditions, performed with the SARS-CoV-2_IBS_RdRP1 primer set. **b** An example of the appearance of short and long primer dimers under a low *T*_a_, with the SARS-CoV-2_IBS_S1 primer set. **c** An example of the appearance of long primer dimers under a low *T*_a_, with the SARS-CoV-2_IBS_E1 primer set. **d** An example of low-efficiency primers in PCR performed with the CDC_RNAse P primer set. **e** An example of the appearance of a nonspecific band at a low *T*_a_, in PCR performed with the SARS-CoV-2_IBS_N1 primer set. Red asterisks indicate long primer dimers.

To demonstrate the importance of the primer optimization procedure, we performed PCR (35 cycles) using various nonoptimal primer sets with or without template DNA (SARS-CoV-2 cDNA) and different *T*_a_ conditions (Fig. 2). The first example demonstrated the appearance of spurious primer–dimer formation (Fig. 2a). A primer dimer is a by-product of PCR, consisting of primer molecules that hybridize with each other because of strings of complementary bases in the primers^22^. In the first example, we performed PCR with the previously reported SARS-CoV-2_IBS_RdRP1 primer set^9^. The resulting electrophoresis data showed the appearance of spurious short dimer bands at approximately 30–50 base pairs (bp), along with a dark band at the expected amplicon size of 118 bp under template conditions (Fig. 2a). In fact, primer–dimer bands were found under all conditions, regardless of the presence of the template. In addition, the intensity of primer–dimer bands tended to decrease as the temperature increased. The appearance of primer–dimer bands suggests that the primer concentration and *T*_a_ are not optimal and need to be optimized by decreasing the primer concentration and increasing *T*_a_.

The second example demonstrated the appearance of spurious long dimer along with a shorter primer–dimer band (Fig. 2b, c). In these examples, we performed PCR with the previously reported SARS-CoV-2_IBS_S1 (Fig. 2b) and SARS-CoV-2_IBS_E1 primer sets (Fig. 2c)^9^. Although there were strong positive bands of the expected size in the conditions including template DNA, long dimer bands appeared at a low *T*_a_ = 50 °C and 55 °C, but not at a high *T*_a_ = 60 °C, in the no-template conditions (red asterisks in Fig. 2b, c). These results suggest that when a primer set shows the potential to form a long PCR by-product, this can be prevented by increasing *T*_a_.

The third example revealed a low-efficiency primer set. In this example, we performed PCR with the previously reported CDC_RNAse P primer set^9^ (Fig. 2d). The resulting electrophoresis data showed that in all conditions, strong spurious long and short primer bands and weak or no expected bands were observed (Fig. 2d). The appearance of these strong primer bands indicated that the primer set presents an excessive dimerization tendency that is stronger than its tendency to hybridize with the template. These results suggest that this primer set should be discarded, and that a new primer set should be designed and optimized.

The last example demonstrated the appearance of a very long nonspecific band. In this example, we performed PCR with SARS-CoV-2_IBS_N1. The resulting electrophoresis data showed no primer–dimer band and a strong expected band in the conditions containing template DNA (Fig. 2e). However, there was a spurious very long nonspecific band near 400 bp at a low *T*_a_ = 50 °C and 55 °C, but not at a high *T*_a_ = 60 °C in the absence of template DNA (Fig. 2e). The absence of a primer–dimer band indicated that the primer set itself was an optimal primer set. However, the presence of a nonspecific band at a low *T*_a_ suggested that the optimal *T*_a_ was higher and was close to *T*_a_ = 60 °C.

In summary, primer sets that show primer–dimer formation can be simply optimized by increasing *T*_a_, whereas the primer set used in Fig. 2d needs to be redesigned. When not optimized, spurious primer dimers and by-products that are formed can lead to the appearance of a false-positive curve at high C_*t*_ values close to 35 cycles and low melting temperatures, regardless of the presence or absence of the template in real-time PCR^23^. When designing a new primer set, one can follow the three-step guidelines that we have proposed in Fig. 1a.

### SARS-CoV-2 primer design and optimization

In this study, we planned to develop a detection protocol for traditional PCR as well as real-time PCR. In addition, we planned to develop a detection protocol for multiplex PCR as well as multiplex real-time PCR. We designed and optimized the primer sets for each protocol according to the guidelines shown in Fig. 1a. The list of the newly designed primer sets as well as the most optimized primer sets from the previous study^9^ are shown in Table 1. Our primer sets were designed specifically for SARS-CoV-2 so that they did not target other human coronaviruses, such as human coronavirus OC43 (HCoV-OC43), human coronavirus NL63 (HCoV-NL63), human coronavirus HKU1 (HCoV-HKU1), and human coronavirus 229E (HCoV-229E).

**Fig. 3:**
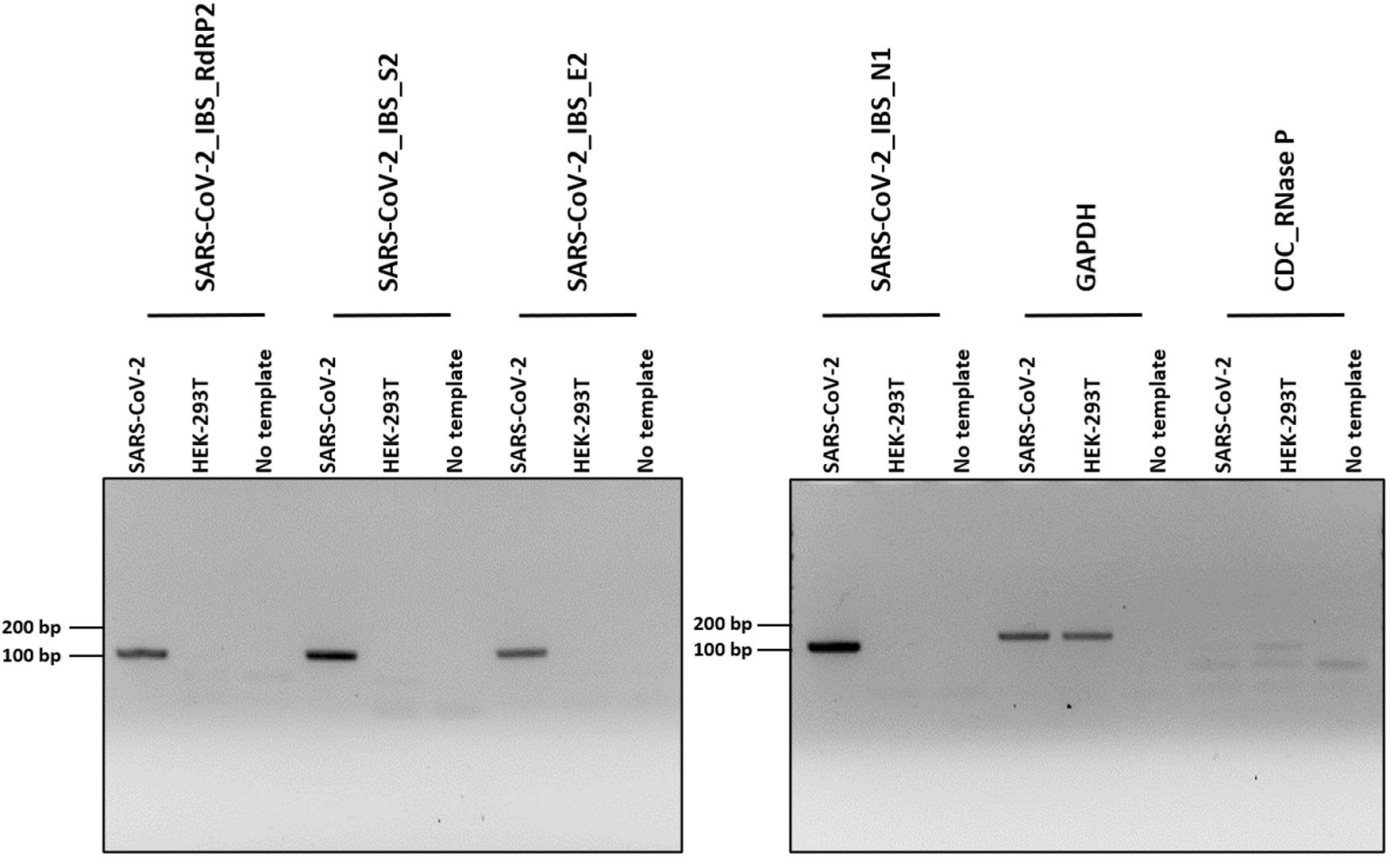
Development of a traditional PCR protocol for SARS-CoV-2 detection. Gel electrophoresis results from PCR (35 cycles) using the primer sets SARS-CoV-2_IBS_E2, SARS-CoV-2_IBS_RdRP2, SARS-CoV-2_IBS_S2, and SARS-CoV-2_IBS_N1. GAPDH primers were used as the IPC primer set. The predicted size of the amplicons was approximately 100 bp. Five nanograms of SARS-Cov-2 cDNA (4.5 × 10^8^ copies/reaction) or HEK-293T cDNA (1.5 × 10^3^ copies/reaction) was used for each reaction. The no-template condition contained no cDNA.

**Fig. 4:**
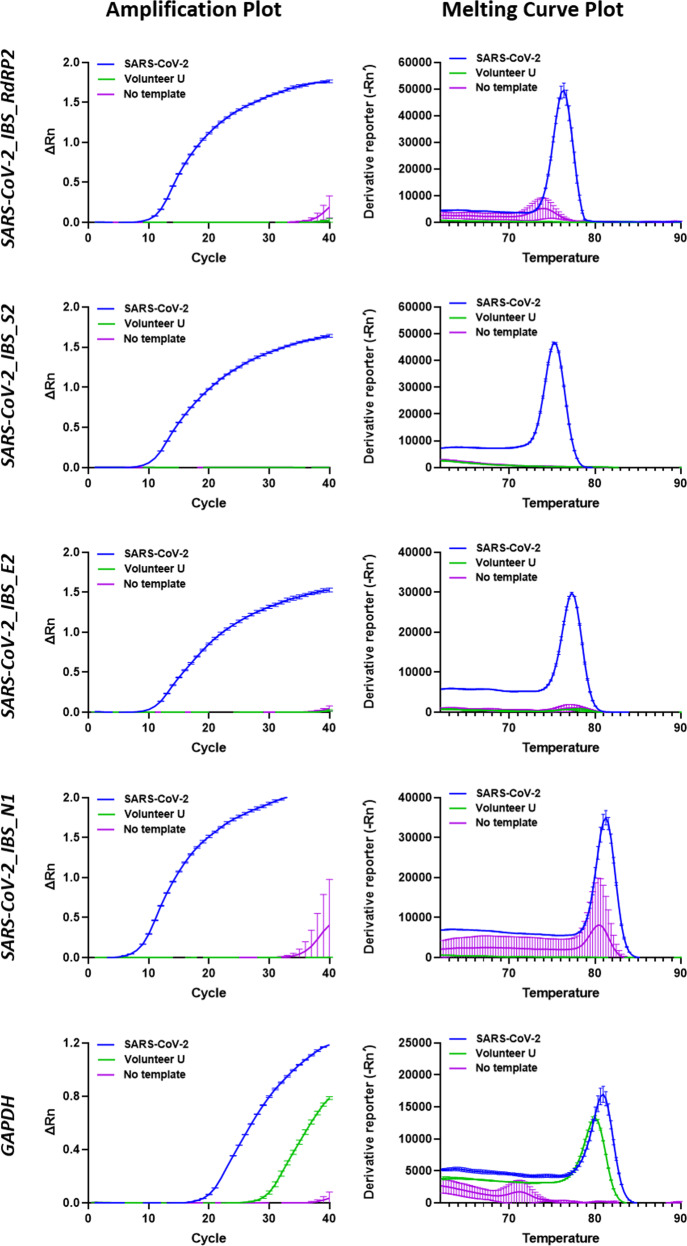
Improvement of the real-time PCR detection protocol for SARS-CoV-2. Real-time PCR was performed using the SARS-CoV-2 primer sets SARS-CoV-2_IBS_E2, SARS-CoV-2_IBS_RdRP2, SARS-CoV-2_IBS_S2, and SARS-CoV-2_IBS_N1. The GAPDH primer set was used as the IPC primer set. Each row represents each primer set. On the left, each graph represents an amplification plot, which shows the variation of log (ΔRn) values against the PCR cycle number. The Y axis represents the normalized reporter value (Rn), which was calculated as the fluorescence signal from SYBR Green normalized to the fluorescence signal of a reference dye. The graphs on the right represent the melting curve plots, which display data collected during a melting curve stage. Peaks in the melting curve may indicate the melting temperature (*T*_m_) of a target or identify nonspecific PCR amplification. On the *Y* axis, the derivative reporter (−Rn′) was calculated as the negative first derivative of Rn generated by the reporter during PCR amplification. The green curve is for the Volunteer U cDNA sample. The blue curve is for the SARS-CoV-2 cDNA. The purple curve is for the no-template condition. All data are represented as the mean ± S.E.M.

**Fig. 5:**
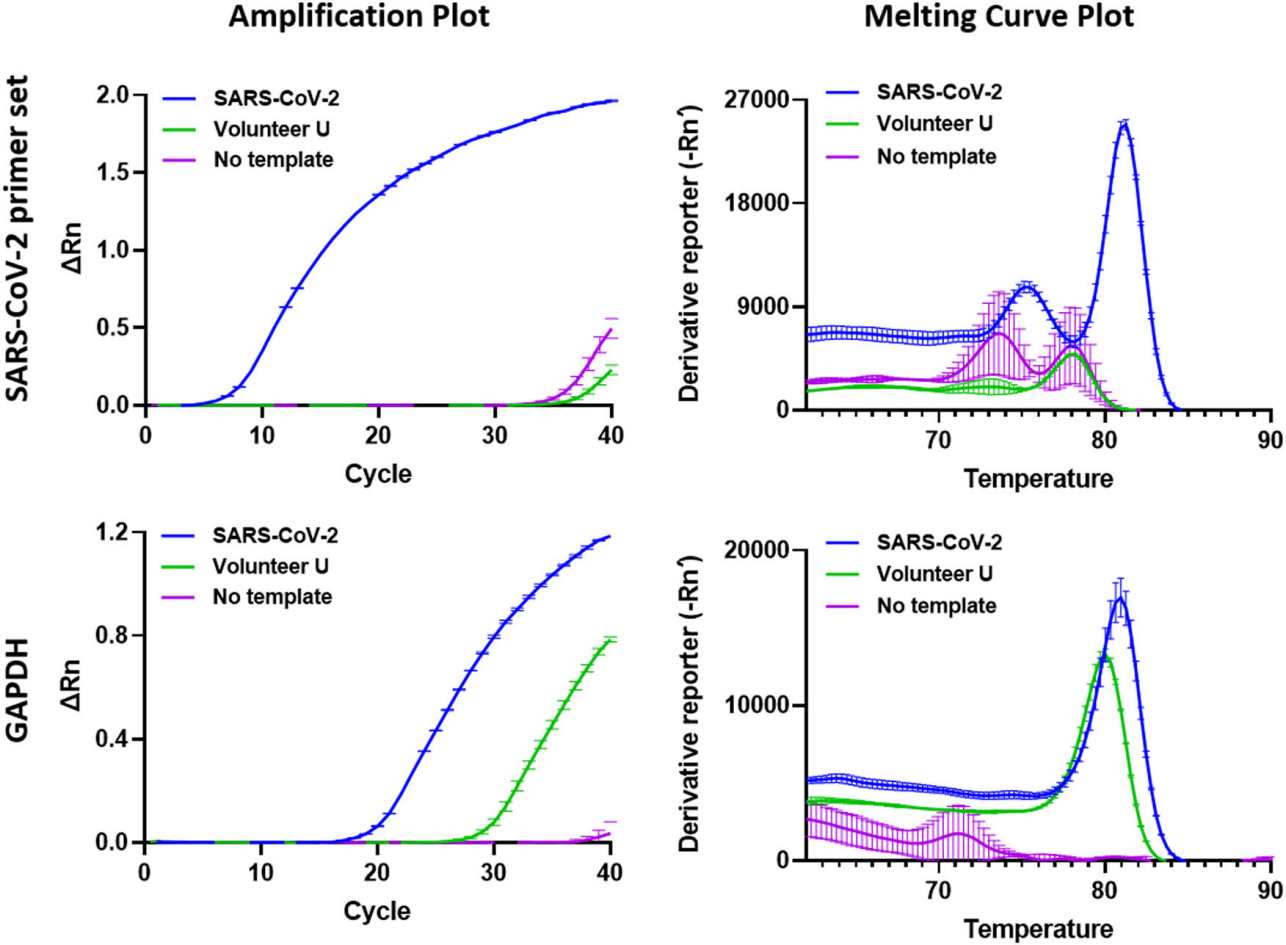
Development of a multiplex real-time PCR detection protocol for SARS-CoV-2. Multiplex real-time PCR results for the SARS-CoV-2 primer sets SARS-CoV-2_IBS_E2, SARS-CoV-2_IBS_RdRP2, SARS-CoV-2_IBS_S2, and SARS-CoV-2_IBS_N1. The GAPDH primer set was used as the IPC primer set. The graphs on the left represent amplification plots. The graphs on the right represent melting curve plots. The final concentration of the primer mix was 500 nM. The green curve is for the Volunteer U cDNA sample. The blue curve is for the SARS-CoV-2 cDNA. The purple curve is for the no-template condition. All data are represented as the mean ± S.E.M.

**Fig. 6:**
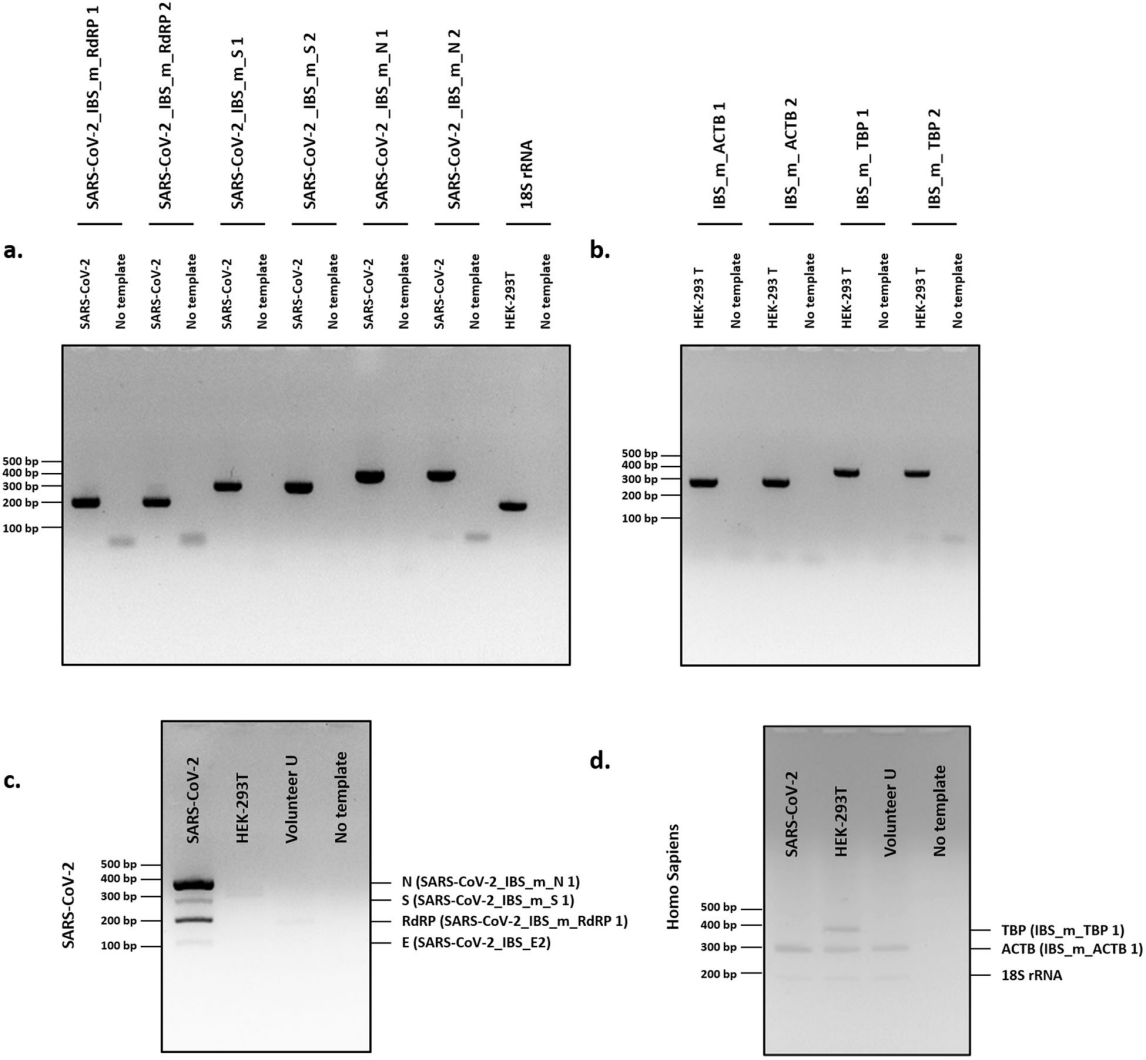
Optimization and development of a multiplex PCR protocol for SARS-CoV-2 detection. **a**, **b** Gel electrophoresis results from PCR with the primer sets SARS_CoV-2_IBS_m_RdRP 1, SARS_CoV-2_IBS_m_RdRP 2, SARS_CoV-2_IBS_m_S 1, SARS_CoV-2_IBS_m_S 2, SARS_CoV-2_IBS_m_N 1, SARS_CoV-2_IBS_m_N 2, 18S rRNA, IBS_m_ACTB 1, IBS_m_ACTB 2, IBS_m_TBP 1, and IBS_m_TBP 2. Expected PCR product sizes are indicated in Table 1. **c**, **d** Gel electrophoresis results obtained from multiplex PCR in the SARS-CoV-2, HEK-293T, Voulteer U, and no-template reactions by using a mixture of four primer sets for **c** SARS-CoV-2 detection (SARS-CoV-2_IBS_E2, SARS-CoV-2_IBS_m_RdRP 1, SARS-CoV-2_IBS_m_S 1, and SARS-CoV-2_IBS_m_N 1). **d** A mixture of three primer sets was used to detect human IPC (IBS_m_TBP 1, IBS_m_ACTB 1, and 18S rRNA). The location of each predicted PCR product in the gel is indicated on the right side.

Among the primer sets that were developed for the real-time PCR-based detection of SARS-CoV-2 in the previous study^9^, some primer sets were re-evaluated and selected on the basis of the design and optimization guidelines presented in Fig. 1a (Table 1). Using the selected primer sets, we performed traditional PCR, as shown in Fig. 3, and real-time PCR, as shown in Figs. 4, 5. Furthermore, the newly designed primer sets produced amplicon sizes ranging from 100 bp to 400 bp. These new primer sets targeted the *RdRP*, *E*, *N*, and *S* genes of SARS-CoV-2 (Fig. 1b, c) and were used for multiplex PCR in Fig. 6 (Table 1). For the detection of the human IPC, we designed new primer sets targeting the *ACTB* (Actin beta), *TBP* (TATA-Box Binding Protein), *18S rRNA*, and *GAPDH* genes of *Homo sapiens* (Table 1). These IPC primer sets were used for the quality control of the volunteered sample cDNA and PCR verification. The new primer sets for multiplex PCR detection were optimized as shown in Fig. 6a, b, according to the guidelines shown in Fig. 1a.

### Development of a traditional PCR protocol for SARS-CoV-2 detection

To develop a traditional PCR detection protocol for SARS-CoV-2 that can be easily implemented in any biological laboratory worldwide, we developed a PCR-based protocol using the best primer sets among the previously developed primer sets^9^ after the optimization of each primer set according to the guidelines shown in Fig. 1a. We used SARS-CoV-2 cDNA as a positive control, human-derived HEK-293T cDNA as the human IPC, and CDC_RNAae P (Fig. 2d) as the primer–dimer control. We performed PCR (35 cycles) for each primer set using Taq polymerase (Fig. 3). The electrophoresis results showed that the SARS-CoV-2 primer sets SARS-CoV-2_IBS_E2, SARS-CoV-2_IBS_RdRP2, SARS-CoV-2_IBS_S2, and SARS-CoV-2_IBS_N1 produced dark bands of the appropriate sizes (~100 bp) only in the presence of the SARS-CoV-2 cDNA template and not in the HEK-293T cDNA or no-template conditions (Fig. 3). The SARS-CoV-2 human IPC primer sets showed few signs of producing spurious primer–dimer bands (Fig. 3), indicating that the primer set was highly optimized compared with the unoptimized primer sets. We used the GAPDH primers as a human IPC primer set and found that although the SARS-CoV-2 primer sets produced no detectable band with HEK-293T cDNA, the GAPDH primer set produced a positive band in the presence of HEK-293T cDNA (Fig. 3). The GAPDH results indicated that the HEK-293T cDNA was properly prepared. Because the SARS-CoV-2 genomic RNA was originally prepared from the Vero cell line, isolated from kidney epithelial cells extracted from an African green monkey (*rhesus macaque*)^19^, the positive GAPDH band obtained in the presence of SARS-CoV-2 cDNA indicated that Vero cell cDNA was inadvertently included in the SARS-CoV-2 cDNA (Fig. 3). This was confirmed with in silico PCR tool as shown in Fig. 1a. Taken together, the results demonstrated that we have developed a traditional PCR-based detection protocol for SARS-CoV-2 that should be feasible to use worldwide in any biological laboratory equipped with a conventional PCR machine.

### Improvement of the real-time PCR detection protocol for SARS-CoV-2

In our previous report, we provided 9 unique primer sets targeting SARS-CoV-2^9^. However, those primer sets had not been optimized according to the guidelines shown in Fig. 1a. Therefore, in this study, we optimized and selected the best primer sets in an attempt to improve the real-time PCR-based protocol. Using the selected primer sets for SARS-CoV-2_IBS_E2, SARS-CoV-2_IBS_RdRP2, SARS-CoV-2_IBS_S2, and SARS-CoV-2_IBS_N1, we performed real-time PCR for each primer set and the Volunteer U or SARS-CoV-2 cDNA sample or the no-template control (Fig. 4). The real-time PCR results are displayed in two separate plots for each primer set: an amplification plot and a melting curve plot (Fig. 4). The amplification plot shows the variation of log (ΔRn) values with the PCR cycle number, whereas the melting curve plot shows the melting dynamics of the dissociation of the DNA strands and the subsequent release of DNA bound with SYBR® Green I dye^24^. The shape and position of this DNA-melting curve are functions of the GC/AT ratio, length, and sequence, and can be used to differentiate amplicons separated by less than 2 °C in melting temperature^24^. The results of the amplification and the melting curve plot for each primer set showed that there was no significantly positive signal at C_t_ values under 37 for all four of the SARS-CoV-2 primer sets (SARS-CoV2_IBS_RdRP2, SARS-CoV2_IBS_S2, SARS-CoV2_IBS_E2, and SARS-CoV2_IBS_N1), whereas there was a positive signal for GAPDH when the Volunteer U sample cDNA was used (green curve, Fig. 4; Supplementary Table 1). This was in great contrast to the significantly positive signals obtained with the SARS-CoV-2 cDNA (blue curve, Fig. 4). The positive signals in the amplification plot were consistently represented by sharp peaks in the corresponding melting curve plot (Fig. 4). The discrepancy between the locations of the peaks in the melting curves of GAPDH for the SARS-CoV-2 and Volunteer U samples was most likely due to the 11% sequence difference in the amplicons between the two primate species (*Homo sapiens* and *rhesus macaque*), as indicated by in silico PCR (Supplementary Table 1). To determine the presence of SARS-CoV-2, we used the same criteria described in the previous paper^9^: if any one of the SARS-CoC-2 genes was detected, it was recorded as “SARS-CoV-2 detected”, or if only the human IPC gene is found, it was recorded as “SARS-CoV-2 NOT detected”. Based on these results and the applied criteria, we concluded that Volunteer U was most likely negative for SARS-CoV-2. Taken together, these results indicate that the use of the optimized primer sets along with melting curve analysis can provide an improved detection protocol for SARS-CoV-2 with no false positives.

### Development of a multiplex real-time PCR protocol for SARS-CoV-2 detection

With the optimized primer sets for real-time PCR, we tried to develop an alternative detection protocol by adopting multiplex real-time PCR, in which all the primer sets are mixed in one reaction buffer. By developing a protocol for multiplex real-time PCR, we expected to reduce the required amounts of the sample and reagents, preparation time, cost, and labor. To obtain the primer set mixture, we mixed all the SARS-CoV-2-targeted primer sets together in reaction buffer, and the IPC primer set was dissolved separately in reaction buffer. Thus, we were able to reduce the total number of reactions from 15 to 6 when we adopted the multiplexing method. To reduce primer–dimer formation when all primer sets were mixed together, we reduced the concentration of each primer set proportionally so that the final concentration of all primer sets was 500 nM (125 nM each). The rest of the procedure was the same as for real-time PCR. We obtained very similar results in the amplification plots for multiplex real-time PCR (Fig. 5) to those obtained via real-time PCR (Fig. 4). In particular, the amplification plot showed one combined reaction curve for all four primer sets with a significantly lower C_t_ value (Fig. 5; Supplementary Table 1) compared with that of each primer set (Fig. 4). The accuracy of the experiment was confirmed by the melting curve plot, as the melting curve for a positive signal showed multiple peaks, with each peak representing each amplicon for the corresponding primer set (Figs. 4, 5; Supplementary Table 1). Based on these results, we concluded that Volunteer U was most likely to be negative for SARS-CoV-2. Taken together, the results demonstrate that the newly developed multiplex real-time PCR protocol can be used for the fast and accurate detection of SARS-CoV-2 as a cost-effective alternative to the real-time PCR protocol.

### Development of a multiplex PCR protocol for SARS-CoV-2 detection

As an alternative to the traditional PCR-based protocol, we further developed a multiplex PCR-based SARS-CoV-2 detection protocol in which all the primer sets were mixed together in reaction buffer. To visualize and separate each amplicon of each primer set, we designed new primer sets targeting the *RdRP*, *S*, and *N* genes in the SARS-CoV-2 genome, where each primer set produced an amplicon of a different size (i.e., 200 bp, 300 bp, and 400 bp, respectively). For the primer set targeting the *E* gene, the previously reported SARS-CoV-2_IBS_E2 primer set was used, producing an amplicon of 116 bp (Table 1). For the human IPC primer sets, we similarly designed new primer sets targeting *18* *S rRNA*, *ACTB*, and *TBP* with amplicon sizes of 200 bp, 300 bp, and 400 bp, respectively. All of the human IPC primer sets included primer sequences shared with the African green monkey (*rhesus macaque*), except for the IBS_m_TBP 1 primer set. To optimize the newly designed primer sets, we performed PCR (35 cycles) with SARS-CoV-2 cDNA as a positive control and human-derived HEK-293T cDNA as a human IPC (Fig. 6a, b). The electrophoresis results showed positive dark bands of the appropriate size and no primer dimers in the presence of SARS-CoV-2 or HEK-293T cDNA, although some primer sets showed produced primer dimers in the no-template conditions (Fig. 6a, b). These results indicated that all primer sets were highly optimized, showing a high amplification efficiency and low tendency of self- or hetero-primer–dimer formation. Based on low or no primer–dimer band formation and a high amplicon band intensity, we selected the following best primer sets for the multiplex PCR-based SARS-CoV-2 detection protocol: SARS_CoV-2_IBS_m_RdRP 1 (*RdRP*), SARS_CoV-2_IBS_m_S 1 (*S*), SARS_CoV-2_IBS_m_N 1 (*N*), 18S rRNA (*18S rRNA*), IBS_m_ACTB 1 (*ACTB*), and IBS_m_TBP 1 (*TBP*).

Using the selected primer sets from the optimization step, we then performed multiplex PCR for SARS-CoV-2 detection. For multiplex PCR, we mixed all the SARS-CoV-2-targeted primer sets together in reaction buffer, and the human IPC primer sets were dissolved separately in reaction buffer, to obtain a final total primer set concentration of 500 nM in both cases. The upper electrophoresis results showed four positive bands at the appropriate size for each SARS-CoV-2 primer set in a single lane in the presence of SARS-CoV-2 cDNA (Fig. 6c). With the exception of the positive band obtained with the SARS-CoV-2_IBS_E2 primer set, the other three primer sets produced high-intensity positive bands (Fig. 6c). This is consistent with the recent report that the expression of the *E* gene in SARS-CoV-2 is very low^18^. In contrast, there was no sign of a positive band in HEK-293T cDNA, Volunteer U cDNA, or no-template conditions (Fig. 6c). Consistent with the results presented in Fig. 6a, b, there was no sign of primer–dimer formation. The electrophoresis results for the human IPC primer sets showed that there was at least one positive band under the Volunteer U, HEK-293T, and SARS-CoV-2 cDNA conditions, but not under the no-template conditions (Fig. 6d), verifying the reliable extraction of RNA from the volunteered sample. Based on these results, we concluded that Volunteer U was most likely to be negative for SARS-CoV-2. Taken together, the results demonstrate that the newly developed multiplex PCR protocol with optimized primer sets can be useful for the fast, easy access, and visually verifiable detection of SARS-CoV-2.

## Discussion

In this study, we developed detailed and practical guidelines for designing and optimizing primer sets in three important steps (Fig. 1a). These guidelines can be utilized not only for SARS-CoV-2 but for any infectious virus emerging in the future. Based on the optimized primer sets, we also developed detection protocols for SARS-CoV-2 utilizing both traditional and real-time PCR. We further extended these protocols for multiplex PCR as well as multiplex real-time PCR. These PCR-based protocols provide multiple options for the selection of detection protocols suitable for various experimental environments, even in the absence of expensive high-specification equipment.

**Fig. 7:**
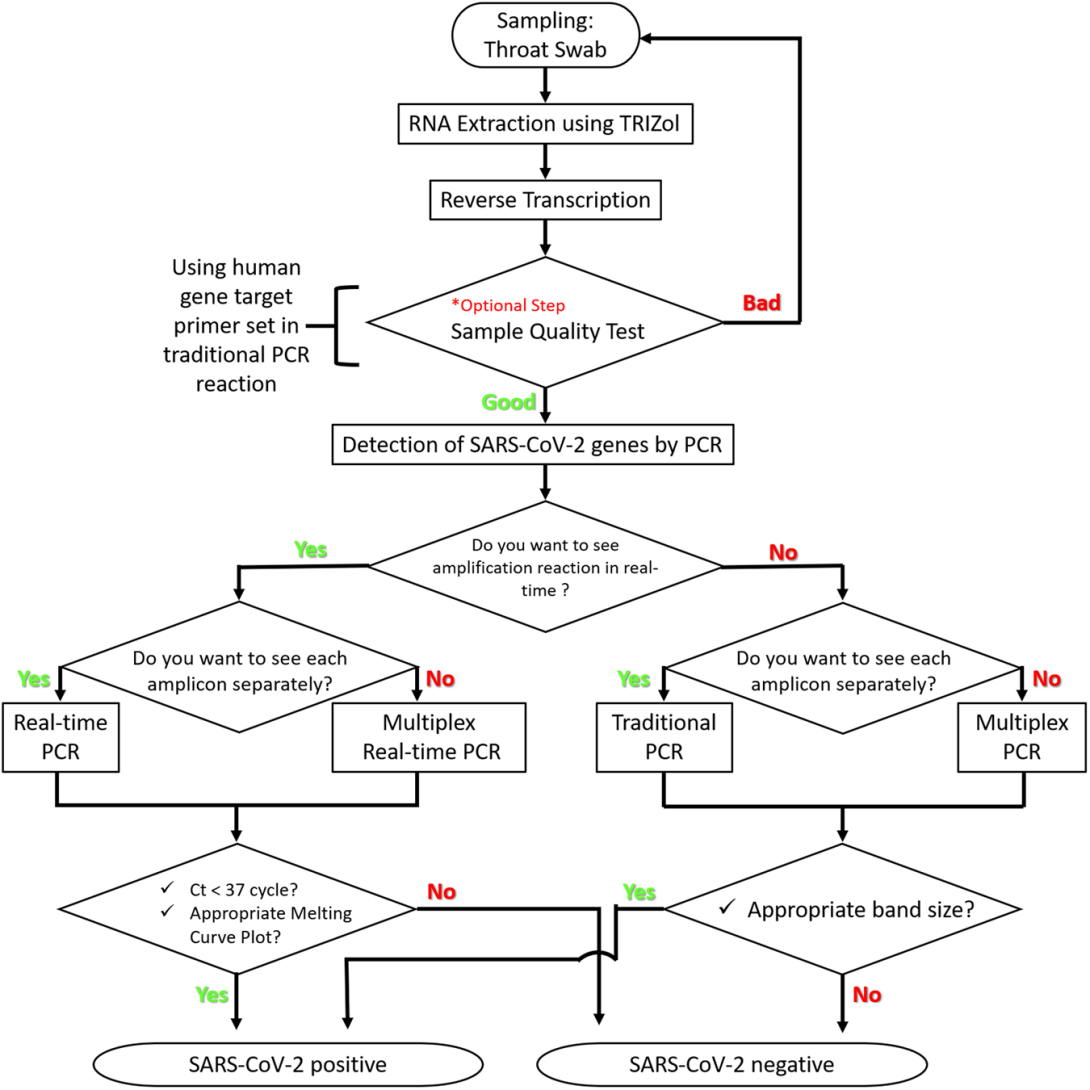
Selection flowchart for SARS-CoV-2 detection protocols. The flowchart represents the sequence of steps and decisions required to perform SARS-CoV-2 detection. The oval symbols indicate the beginning and end of a program or sub-process. The arrows are the way the logic flows. The rectangle symbols represent a set of operations that changes the value, form, or location of data. The diamond symbols show a conditional operation that determines which one of the two paths the program will take. The red asterisk represents an optional sample-quality test step.

These multiple alternatively SARS-CoV-2 detection protocols are summarized in Fig. 7 in a flowchart to visually represent the sequence of steps and decisions required to perform SARS-CoV-2 detection. First, a sample was obtained from each volunteer via a throat swab, which is then subjected to the extraction of total RNA with the TRIzol reagent, which is then converted into cDNA via reverse transcription. Before proceeding to the detection of SARS-CoV-2 genes by PCR, an optional sample-quality test (red asterisk in Fig. 7) is introduced to confirm the quality of the extracted RNA and converted cDNA by utilizing the human IPC primer sets. If the sample quality is not adequate, a user should return to the sampling step and perform resampling. This step should prevent any waste of time and labor due to inadequate RNA or cDNA. In the next step, a user can decide upon the actual detection protocol based on the question “Do you want to see the amplification reaction in real time?” If the answer is “Yes”, a user has selected the protocols based on real-time PCR. If “No,” a user has selected the traditional PCR-based protocol. At the next question in each choice option, “Do you want to see each amplicon separately?”, a user decides upon the option for multiplex PCR. If the answer is “No”, a user decides to proceed with a multiplex-based protocol. After performing PCR, a user determines whether each volunteered sample is positive or negative for the SARS-CoV-2 virus based on the size and intensity of each amplicon band under the traditional PCR-based protocols or the C_t_ value (C_t_ < 37 cycles) and melting curve plot under the real-time PCR-based protocols (Fig. 7).

When a user decides upon a traditional or real-time PCR-based protocol, it is important to consider the advantages and disadvantages of each type of protocol. Real-time PCR-based protocols have the advantages of allowing not only the visualization of the amplification reaction in real time, but also its verification through a melting curve plot, whereas the disadvantages include the user’s inability to visualize the appearance of spurious primer dimers. Traditional PCR-based protocols present the advantages of convenience and cost-effectiveness, requiring a less-expensive conventional PCR machine than a real-time PCR machine, whereas the disadvantages include the user’s inability to automate due to the requirement of gel electrophoresis. Another advantage includes the user’s ability to visualize spurious primer–dimer formation.

In general, multiplex PCR (either traditional or real time) has advantages of fast screening, accurate detection, and cost-effectiveness, whereas the disadvantages of this approach include the possibility that the mixture of multiple primer sets will affect the amplification process because of competition between primer sets for limited resources such as polymerases and deoxynucleotide triphosphates (dNTPs). Real-time PCR results can reveal the accuracy of each amplicon based on melting curve analysis, whereas multiplex real-time PCR results often cannot reveal the accuracy of each amplicon due to overlapping *T*_m_ values of multiple amplicons. In our results, multiplex real-time PCR producing the RdRP, S, E, and N amplicons produced similar predicted *T*_m_ values (Supplementary Table 1; Fig. 4), and only two, rather than four, peaks appeared in the meting curves (Supplementary Table 1; Fig. 5). Although the peak values were within the predicted *T*_m_ range (Supplementary Table 1), it was impossible to separate the peak values for each amplicon in the melting curve (Fig. 5). To overcome this limitation, we introduced a multiplex PCR protocol that allows the clear distinction of each amplicon in a single gel (Fig. 6c). These considerations should help a user in making a wise selection regarding the most suitable SARS-CoV-2 detection protocol.

In our previous study, we identified critical issues related to unoptimized primer sets leading them to inadvertently show false-positive results^9^. There are many commercially available kits and government-supplied kits with undisclosed primer set sequences. These results raise the dismal possibility that some of these diagnostic kits whose primers are not disclosed might contain unoptimized primer sets that produce false-positive results. In the current study, we found numerous examples of unoptimized primer sets producing long and short dimer bands (Fig. 2), which could be a potential source of false-positive results in real-time PCR- and traditional PCR-based protocols. Consistent with this possibility, it has been reported that primer dimers can be unexpectedly observed in gel electrophoresis and melting curve analyses with SYBR Green dye^25^. Therefore, we strongly recommend that the critical primer sets in PCR-based SARS-CoV-2 diagnostic kits should be disclosed publicly and optimized according to our guidelines.

In summary, our study provides the first guideline for SARS-CoV-2 detection protocols. In addition, we have fully disclosed the optimized primer sets targeting the four essential genes of SARS-CoV-2. In combination with the information available from our previous study^9^, anyone with basic knowledge of biology should be able to develop an easy-access, low-cost, fast, flexible, and high-accuracy detection kit for SARS-CoV-2. We hope that our efforts will be helpful for establishing a large-scale, high-fidelity screening approach for asymptomatic people for the further prevention of transmission and early intervention and treatment for rapidly propagating COVID-19.

## Supplementary information

Supplementary Table.1 Real-time and Multiplex real-time PCR results
