# Supplementary material for: Optimization of primer sets and detection protocols for SARS-CoV-2 of coronavirus disease 2019 (COVID-19) using PCR and real-time PCR
Source: Exp Mol Med. 2020 Jun 16;52(6):963–77. doi: 10.1038/s12276-020-0452-7 (PMC7295692; doi:10.1038/s12276-020-0452-7)
Supplement: Supplementary file 1 — Supplementary Table.1 Real-time and Multiplex real-time PCR results [file 12276_2020_452_MOESM1_ESM.docx]

**Supplementary Table.1 Real-time and Multiplex real-time PCR results**

Real time PCR (Fig. 4) and multiplex real-time PCR (Fig. 5) results. Expected T_m_ calculated by Oligo Calc: Oligonucleotide Properties Calculator tool in website (<http://biotools.nubic.northwestern.edu/OligoCalc.html>) based on each amplicon sequence. SARS-CoV-2 primer sets contain SARS-CoV-2_IBS_E2, SARS-CoV-2_IBS_RdRP2, SARS-CoV-2_IBS_S2 and SARS-CoV-2_IBS_N1. All data are represented as mean ± S.E.M. u.d. means undetermined data.

**Supplementary Table.1**

| **Primer name** | **Experiment** | **Sample** | **Ct** | **Expected T_m_** | **T_m_1** | **T_m_2** |
| --- | --- | --- | --- | --- | --- | --- |
| SARS-CoV-2_IBS_RdRP2 | Real-time  PCR | SARS-CoV-2 | 10.1± 0.00 | 75.2 | 76.3± 0.08 |  |
|  |  | Volunteer U | u.d |  | 62.0± 0.00 |  |
|  |  | No template | 37.5± 0.81 |  | 74.2± 0.23 |  |
| SARS-CoV-2_IBS_S2 |  | SARS-CoV-2 | 10.8± 0.00 | 74.6 | 77.3± 0.00 |  |
|  |  | Volunteer U | u.d |  | 77.4± 0.76 |  |
|  |  | No template | u.d |  | 77.1± 7.57 |  |
| SARS-CoV-2_IBS_E2 |  | SARS-CoV-2 | 10.0± 0.02 | 76.4 | 75.3± 0.00 |  |
|  |  | Volunteer U | u.d |  | 85.7± 5.35 |  |
|  |  | No template | 39.8 |  | 87.7± 3.98 |  |
| SARS-CoV-2_IBS_N1 |  | SARS-CoV-2 | 8.4 ± 0.03 | 80.4 | 81.3± 0.00 |  |
|  |  | Volunteer U | u.d |  | 62.2± 0.15 |  |
|  |  | No template | 35.5 |  | 71.3± 9.26 |  |
| GAPDH |  | SARS-CoV-2 | 19.9± 0.04 | 79.8, 80.8 | 81.0± 0.00 |  |
|  |  | Volunteer U | 29.5± 0.28 |  | 80.0± 0.00 |  |
|  |  | No template | 39.7 |  | 66.6± 4.59 |  |
| SARS-CoV-2 primer sets | Multiplex Real-time PCR | SARS-CoV-2 | 6.5± 0.01 | - | 81.1± 0.00 | 75.3± 0.00 |
|  |  | Volunteer U | 36.8± 0.21 | - | 78.1± 0.00 | 73.3 |
|  |  | No template | 34.9± 0.19 | - | 75.8± 2.22 | 75.8± 2.29 |
